# Adipose tissue derived stem cell secretome induces motor and histological gains after complete spinal cord injury in *Xenopus laevis* and mice

**DOI:** 10.1177/20417314231203824

**Published:** 2024-02-09

**Authors:** Rita C Assunção-Silva, Andreia Pinho, Jorge R Cibrão, Inês M Pereira, Susana Monteiro, Nuno A Silva, Jonas Campos, Ana L Rebelo, Gerhard Schlosser, Luisa Pinto, Abhay Pandit, António J Salgado

**Affiliations:** 1 Life and Health Sciences Research Institute (ICVS), School of Medicine, University of Minho, Braga, Portugal; 2 ICVS/3B’s - PT Government Associate Laboratory, Braga/Guimarães, Portugal; 3 BnML – Behavioral and Molecular Lab, Braga, Portugal; 4 CÚRAM, SFI Research Center for Medical Devices, National University of Ireland, Galway, Ireland

**Keywords:** Spinal cord injury, adipose tissue derived stem cell, secretome, neuroinflammation, axonal growth, regeneration, *Xenopus laevis*, mouse model

## Abstract

Mesenchymal stem cell-based therapies have been studied for spinal cord injury (SCI) treatment due to their paracrine action upon damaged tissues. MSCs neuroregenerative role may relate to the contents of their secretome in anti-inflammatory cytokines and growth-permissive factors. We propose using the secretome of MSCs isolated from the adipose tissue—adipose tissue-derived stem cells (ASCs) as a cell-free based therapy for SCI. In vivo studies were conducted in two SCI models, *Xenopus laevis* and mice, after complete spinal cord transection. Our results on both models demonstrated positive impacts of ASC secretome on their functional recovery which were correlated with histopathological markers of regeneration. Furthermore, in our mice study, secretome induced white matter preservation together with modulation of the local and peripheral inflammatory response. Altogether, these results demonstrate the neuroregenerative and potential for inflammatory modulation of ASC secretome suggesting it as a good candidate for cell-free therapeutic strategies for SCI.

## Graphical abstract

**Graphical abstract:**
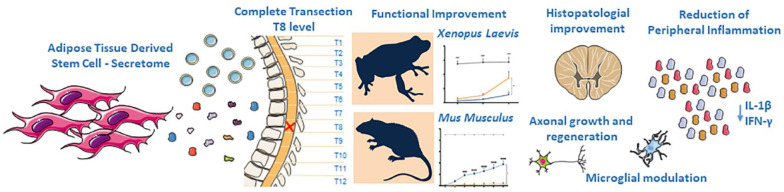

## Introduction

The human spinal cord’s inability to regenerate after injury is due to the extremely complex pathophysiology of spinal cord injury (SCI). The currently available clinical intervention to SCI patients is limited to the decompression/stabilization of the spine^1–3^ and control over possible clinical complications through cardiovascular, respiratory, and circulatory support^3–5^ as well as a pharmacological intervention.^6^ All of these aims to minimize the primary injury, and prevent the secondary injury known to exacerbate the condition.^7^ Despite the progress in these surgical, clinical, and pharmacological approaches, they still do not significantly improve SCI patients’ sensory and motor outcome.^8^ The main mechanisms related to the secondary SCI are neuronal death, inflammation, reactive gliosis, axonal demyelination, and cyst formation, as shown by animal and human studies.^9–16^ Furthermore, the accumulation of inhibitory molecules and loss of trophic support in the lesion site are significant contributors to the limited regeneration of the injured tissues. Despite the development of several strategies, mostly at a pre-clinical stage, focused on controlling or reverting these SCI-related mechanisms, they are yet to be successful, which poses an urgent need to find new SCI strategies treatment.

The two main therapeutic paradigms for SCI rely on the modulation of the inflammatory response after injury, avoiding the exacerbated secondary injury (neuroprotection), or through the replacement of the damaged neural tissue while stimulating the endogenous neural regeneration (neuroregeneration).^7,17^ In this context, stem cell therapies have been a key element in developing strategies that could restore spinal cord function after injury.^7,18^ Nevertheless, these cells’ interest has now turned to their paracrine activity on the damaged tissues rather than looking to their local effect.^19^ This has been due to the perception that some stem cell populations secrete a cocktail of biomolecules to the external milieu, which were considered the main contributors to their neuroregenerative actions.^20–25^ One of the best examples of a stem cell population with a therapeutic impact mediated by the neuroprotective and neuroregenerative character of their secretome are mesenchymal stem cells (MSCs). Their secretome is known to contain important proteins, growth factors, chemokines and cytokines that mediate regeneration.^19^ Indeed, Nerve growth factor (NGF), Vascular endothelial growth factor (VEGF), Hepatocyte growth factor (HGF), Insulin-like growth factor 1 (IGF-1), Transforming growth factor-beta 1 (TGF-β1), Interleukin (IL)-10, Glial-derived neurotrophic growth factor (GDNF), basic Fibroblast growth factor (bFGF), Pigment epithelium-derived factor (PEDF), Cadherin 2 (CADH2), Semaphorin 7A (SEM7A), and Glial-derived nexin (GDN) are some of the factors that have been pointed out as responsible for ASCs neurotrophic and immunomodulatory effects. All of them have been previously related with neurogenesis, neuronal differentiation and proliferation,^26–29^ and axonal growth and migration.^30–35^ From the different available sources to isolate cells with a MSC phenotype, those obtained from the adipose tissue, known as Adipose Tissue derived Stem Cells (ASCs) have shown to be particularly interesting for SCI applications. Indeed, we have previously shown that the secretome of ASCs has a predominant role in promoting neuronal differentiation of human neural progenitor cells and axonal growth of dorsal root ganglion explants, in vitro,^36^ when compared to the secretome of ASCs from other sources such as the bone marrow or umbilical cord tissue. These outcomes have been further attributed to the composition of ASC secretome in several identified central nervous system (CNS)-related neuroregulatory factors, namely Pigment epithelium-derived factor (PEDF), Semaphorins (SEM), Cadherins (CDH), Interleukin (IL)-6, Glial-derived nexin (GDN), Clusterin (CLUS), Decorin (DCN) and Beta-1,4- galactosyltransferase 1 (β4Gal-T1).^36,37^

In recent years, our lab has focused on the potential use of the secretome of stem cells, particularly those with a mesenchymal phenotype, for CNS regenerative purposes. In our view, the use of secretome per se presents numerous advantages compared with more conventional stem-cell based applications, regarding manufacturing, storage, handling, their potential as a ready-to-use biologic product and lack of immunosuppression-based adjuvant therapies.^38^ For instance, the time and cost of expanding and maintaining cultured ASCs could be significantly reduced. The storage can be done for long periods without losing product potency and quality.^38–40^ The production in large quantities is possible under controlled laboratory conditions and the biological product could be modified to desired cell-specific effects.^38,41^ Importantly, the use of secretome derivatives could bypass potential issues associated with cell transplantation including the number of available cells for transplantation and their survival after this procedure, immune compatibility, tumorigenicity, and infection transmission.^42^

Having this in mind, in the present study, we propose the use of the secretome of ASCs isolated from adipose tissue as a cell-free based therapy for SCI. In vivo studies were conducted in two models of SCI—*Xenopus laevis* and mice—both of which showed enhanced neuronal and axonal growth compared to control groups and a strong recovery on the motor and histological parameters.

## Results

### ASC secretome improves functional recovery of Xenopus laevis tadpoles after SCI

**Figure 1.**
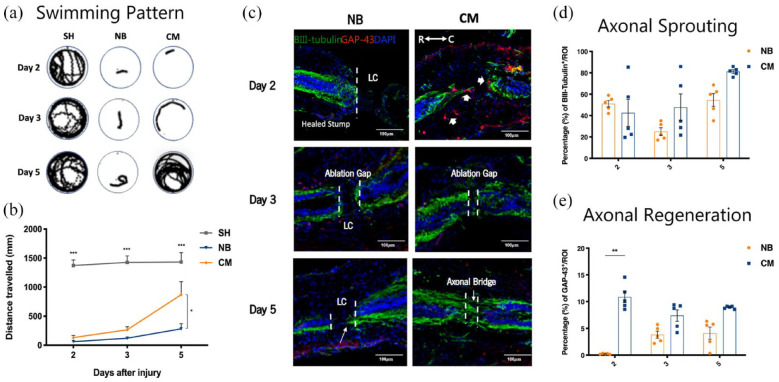
Therapeutic effects of ASC secretome on *Xenopus laevis* tadpoles after complete transection on swimming recovery, axonal growth and regeneration, in the refractory stage of development. (a and b) Swimming pattern and quantification of the distance traveled by the refractory animals after SCI, at 2, 3, and 5 days after treatment with ASC secretome (CM group) or neurobasal medium (NB). Tadpoles with no SCI and treated with neurobasal medium (SH group) were used as controls for healthy animals. ASC secretome promoted significant functional recovery (868.861 ± 223.901 mm) of *Xenopus laevis* tadpoles after SCI from the refractory period, 5 days post-treatment, compared to neurobasal-treated animals (NB group; 283.015 ± 88.471 mm). (c) Representative confocal images of longitudinal cross sections of *Xenopus laevis* spinal cord after immunostaining for βIII-tubulin (axonal sprouting) and GAP-43 (axonal regeneration), at the refractory stage. Quantification of the percentage of (d) βIII-tubulin and (e) GAP-43 immunoreactivity in the spinal cord of Xenopus laevis tadpoles, at the refractory stage. At this stage, the ASC secretome group (CM) showed a clear gap closure and the formation of a robust axonal bridge at the lesion core (LC), between the two stumps of the spinal cord, 5 days post-treatment. This was confirmed by elevated, but not statistically significant, expression of βIII-tubulin in CM group (81.171 ± 1.679) when compared to NB group (54.529 ± 6.015). GAP-43 positive regenerating cells were present in the spinal cord tissue of these animals, with significant differences observed at 2 days post-treatment (CM group: 10.879 ± 1.071 vs NB group: 0.219 ± 0.037). Mean ± SEM; *n* = 12 for locomotor assessment; *n* = 5 for histological evaluation *p < 0.05; **p < 0.01; ***p < 0.001.

In this study, the potential of the ASC secretome in promoting Xenopus laevis spinal cord regeneration after injury was evaluated in the refractory periods, a period (from stage 46 to 47) in which Xenopus larvae, which otherwise are capable to regenerate their spinal cord after injury, temporarily are unable to do so [47]. The motor recovery of tadpoles in response to treatment was assessed by monitoring the animals’ free-swimming ability using a motion capturing software, at 2, 3, and 5 days post-injury. In both periods, paralysis of all animals was observed during the 2 initial days post-treatment. On the following days, the ASC secretome-treated group showed a swimming pattern very similar to non-injured animals (SH group, Figure 1(a)). Significant differences in the swimming distances between the ASC secretome-treated and NB-treated groups were found 5 days post-treatment with (868.861 ± 223.901 mm) in the CM group versus (283.015 ± 88.471 mm) in the NB group as witnessed in Figure 1(b); *p < 0.05.

### ASC secretome favors axonal sprouting and regeneration in Xenopus laevis tadpoles after SCI

After treatment, neuronal regrowth and regeneration were assessed by performing βIII-tubulin and GAP-43 immunostaining, respectively, at 2, 3, and 5 days post-injury for refractive period animals. Substantial GAP-43 expressing cells in the lesion core (LC) at both rostral and caudal ends of the spinal cord was observed in the ASC secretome-treated group (CM, Figure 1(c), arrow heads), 2 days after treatment, but few were observed for the NB-treated group (NB, Figure 1(c)). This was confirmed by significant differences in the mean percentage of GAP-43^+^ cells between the ASC secretome-treated group (10.879 ± 1.071) and the NB-treated group (0.219 ± 0.037) (**p < 0.01, Figure 1(e)). Considerable ablation gap closure and a robust axonal bridge formation was also observed in the secretome-treated animals, 3 and 5 days after treatment, respectively (CM, Figure 1(c)). Furthermore, a tendency for increased expression of βIII-tubulin in the LC of tadpole’s spinal cord, 5 days post-treatment, indicated neuronal regrowth throughout the injury site (CM, Figure 1(d)), CM group (81.171 ± 1.679) when compared to NB group (54.529 ± 6.015), though no statistical differences were found between groups.

### ASC secretome improves motor function of mice after SCI

**Figure 2.**
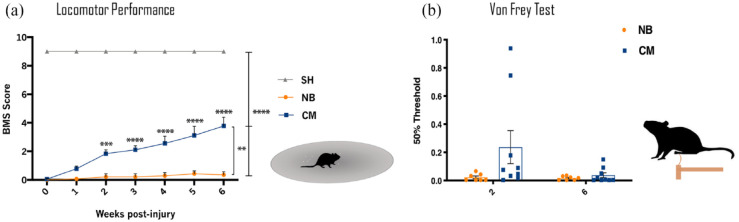
Recovery of motor and sensorial function of mice with complete spinal cord transection after ASC secretome treatment. (a) BMS test was performed up to 6 weeks after treatment. ASC secretome treatment (CM) significantly improved the locomotor function of the transected animals, when compared to NB treatment CM-group at 6 weeks (3.778 ± 0619) versus NB-group (0.357 ± 0.210). Animals with no SCI treated with NB medium (SH group) showed completely normal locomotor performance. (b) Von-Frey Trial was performed at 2 and 6-week after ASC secretome or NB treatment as measure of sensitivity regain. Although no statistical differences were found between groups at both time-points, CM group show a trend of recovery of the sensorial function (0.237 ± 0.351) early at 2 weeks post-injury, when compared to NB group (0.022 ± 0.025). Data is presented as Mean ± SEM; *n* = 8 (SH), *n* = 7 (NB), *n* = 9 (CM); **p < 0.01; ***p < 0.001; ****p < 0.0001.

Functional recovery was assessed weekly using the BMS score for a total of 6-weeks, as depicted in Figure 2(a). Two days post-injury, all SCI animals presented complete paralysis of the hindlimbs, when compared to laminectomy animals (SH group). In the following weeks, ASC secretome-treated animals showed a gradual recovery of hindlimb movements for up to 6 weeks. The NB-treated animals only showed the limited and slight movement of one or two joints. The improvement of the secretome treated group was found to be significantly higher CM-group (1.833 ± 0.276) already at 2 weeks post-treatment in comparison to the NB-group (0.214 ± 0.214) (*** *p* < 0.001), and persisted to increase from 3 to 6 weeks (**** *p* < 0.0001). At 6 weeks post-treatment, secretome-treated animals showed the ability to frequently or consistently perform plantar stepping, accompanied by some degree of coordination. CM-group at 6 weeks (3.778 ± 0619) versus NB-group (0.357 ± 0.210).

The motor function improvements of the secretome-treated SCI mice were accompanied by an apparent sensory recovery of the hindlimbs (Figure 2(b)), as NB-treated animals seem to present lower magnitude of response to the Von Frey filaments than secretome-treated animals, at both time points. Healthy individuals usually present a high threshold of response, indicating normal sensitivity to the mechanical stimuli, while low threshold indicates hypersensitivity.^43^

### ASC secretome modulates neuroinflammation in mice after SCI

**Figure 3.**
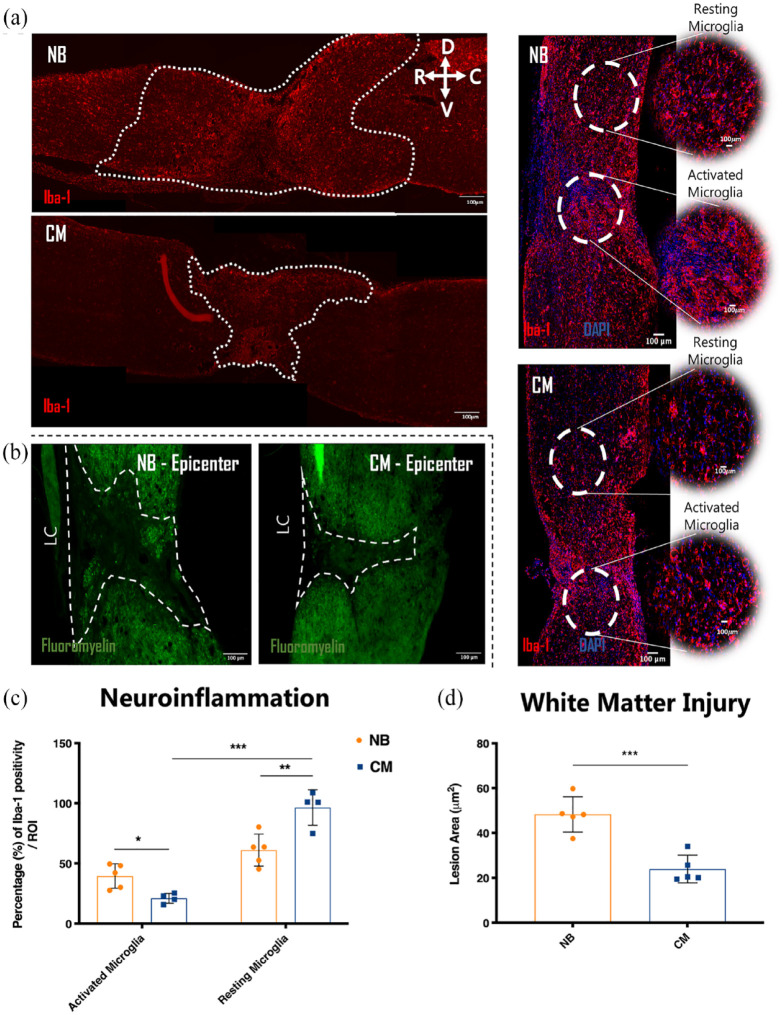
Therapeutic effects of ASC secretome in mice spinal cord 6 weeks after complete transection on neuroinflammation and white matter injury. (a–d) Representative confocal images of longitudinal cross sections of mice spinal cord after immunostaining for Iba-1 ((a), neuroinflammation), fluoromyelin (b), white matter injury. Quantification of the (c) percentage of Iba-1 reactivity, (d) area of injured white matter. ASC secretome group (CM) presented statistically significant effect in the modulation of microglial response, decreasing the area of inflammatory microglial cells (21.124 ± 2.060) while increasing the area of homeostatic ones (96.396 ± 7.370) when compare to NB group (39.554 ± 4.536) and (61.105 ± 5.944), respectively. Similar effects were produced toward the area of injured white matter as CM-group had lower (23.933 ± 2.764), in comparison to the (NB) (39.554 ± 4.536) white matter degeneration. Dashed Lines denotes area coverage of ameboid microglia in (a) and epicenter regions in (b). Data is presented as Mean ± SEM; *n* = 5; *p < 0.05; **p < 0.01; ***p < 0.001.

The inflammatory response following injury was clearly different between the secretome and NB groups, as suggested by the Iba-1 staining. Ratios of the areas occupied by surveiling versus reactive microglia were quantified along the rostro-caudal axis of the spinal cord tissue (Figure 3(a)). Different distributions in the areas of surveiling and reactive cells were shown in the two groups, with prominent round-shape reactive cell accumulation beyond the lesion site for the NB group, as outlined in Figure 3(a) (dashed lines). Quantification of the percentage of Iba-1 reactivity confirmed the significantly higher levels of inflammatory cells in the NB-group (39.554 ± 4.536) when compared to the CM-group (21.124 ± 2.060) (Figure 3(c); *p < 0.05) and lower resting ones (Figure 3(c); **p < 0.01) NB-group (61.105 ± 5.944) versus CM-group (96.396 ± 7.370), respectively.

### ASC secretome preserves white matter in mice after SCI

The border of the lesion area, shown in Figure 3(b) (dashed line), was clearly outlined by fluoromyelin staining. Larger cavities were found for the NB group (Figure 3(b), NB), when compared to the secretome-treated group (Figure 3(c), CM), further confirmed by the quantification of the preserved myelinated area (Figure 3(d)) 6 weeks after treatment with NB-group (39.554 ± 4.536) and CM-group (23.933 ± 2.764) (*** *p* < 0.001).

### ASC secretome promotes neurite regeneration and sprouting in mice spinal cord after injury

**Figure 4.**
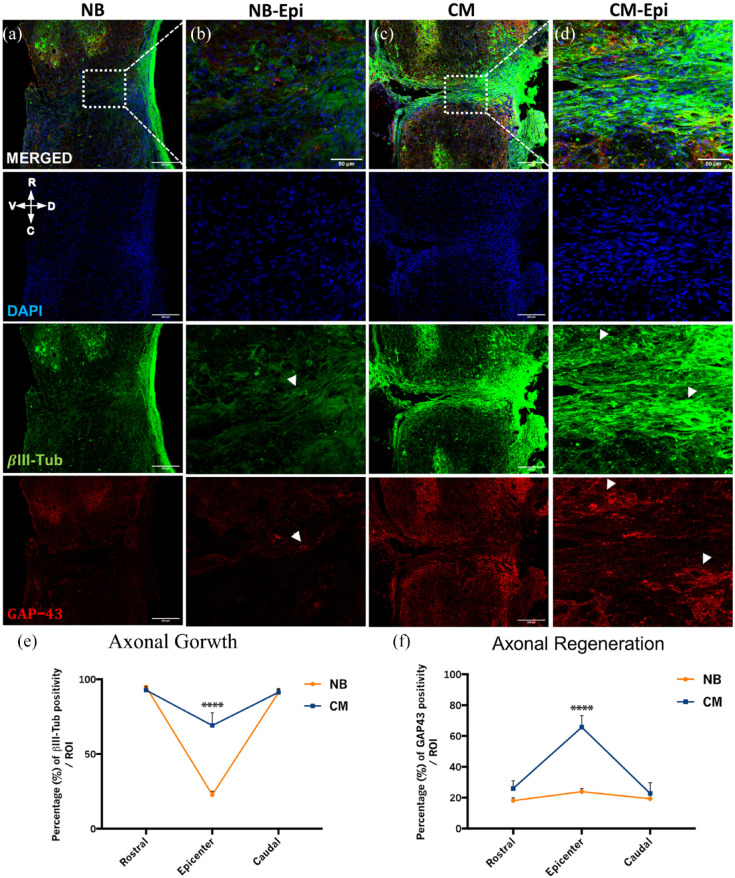
Therapeutic effects of ASC secretome in mice spinal cord 6 weeks after complete transection on axonal growth and regeneration. (a–e) Representative confocal images of longitudinal cross sections of mice spinal cord after immunostaining for ßIII-tubulin (axonal growth) and GAP-43 (axonal regeneration). Axonal outgrowth at the lesion site, as measured by a significant increase of ßIII-tubulin for ASC secretome group (69.106 ± 19.013), in comparison to NB group (22.677 ± 5.535) (e). Regeneration fibers were also clearly observed in CM group, as denoted by a significant increase GAP-43 expression at the lesion site (65.695 ± 16.990), when compared to NB group (23.193 ± 4.423) (f). Data is presented as Mean ± SEM; *n* = 5; ****p < 0.0001. White arrows point to ßIII-tubulin and GAP-43 immuno reactive fibers in (b and d) single channel images respectively. Scale bars are 200 and 50 µm in (a, c) and (b, d) respectively.

Axonal sprouting and regeneration were evaluated 6 weeks after treatment by βIII-tubulin and GAP-43 immunoreactivity in mice spinal cord tissue, respectively. Immunohistochemistry analysis of the spinal cord tissue of ASC secretome treated animals showed βIII-tubulin^+^ axons sprouting from the stumps of the spinal cord into the lesion area (epicenter) (Figure 4(c), CM-epicenter and close-up image and arrows, Figure 4(d)). In contrast, the NB group presented a significantly smaller density of βIII-tubulin ^+^ axons in the proximities of the epicenter (Figure 4(a), NB-epicenter and close-up image and arrow, Figure 4(b)). These observations were further confirmed by the significant differences on the percentage of βIII-tubulin positive cells in the epicenter between the two groups CM-group (69.106 ± 19.013) versus NB-group (22.677 ± 5.535) (Figure 4(e); ****p < 0.0001). Moreover, regenerating GAP-43^+^ axons were found extending through the lesion area in the ASC secretome treated animals (Figure 4(c); CM-epicenter and close-up image and arrows, Figure 4(d)), while the NB-treated animals only presented few of them surrounding the lesion site (Figure 4(a), NB-epicenter and close-up image and arrow, Figure 4(b)). Some GAP-43^+^ axons were also found rostrally and caudally to the lesion area in the secretome-treated group (Figure 4(b), CM), but few were seen in the NB-group (Figure 4(b), NB). Accordingly, significant differences were obtained in the percentage of GAP-43 labeling in the epicenter between groups CM-group (65.695 ± 16.990) versus NB-group (23.193 ± 4.423) (Figure 4(f); ****p < 0.0001).

### ASC secretome provides an anti-inflammatory effect in mice after SCI

**Figure 5.**
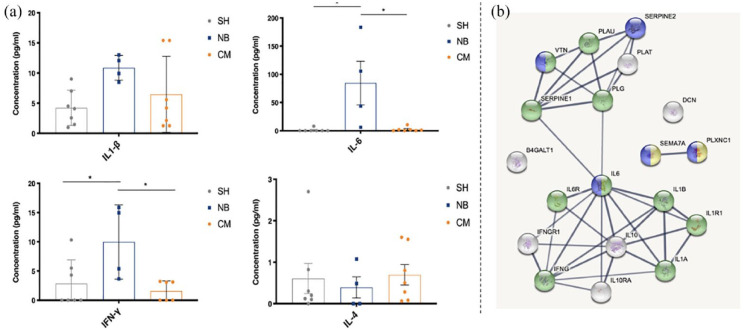
Concentration of cytokines in the blood serum of mice at 6 weeks post-injury, and their interaction with other molecules present in ASC secretome. (a) The concentration (pg/ml) of pro-inflammatory—IL-1β, IL-6, IFN-γ and anti-inflammatory—IL-4 cytokines in the blood serum of SCI mice was assessed using multiplex-based ELISA. Secretome group (CM) showed decreased levels of pro-inflammatory cytokines (IL1-β: 6.463 ± 2.383; IL-6: 2.286 ± 1.460; IFN-γ: 1.573 ± 0.704), and increased levels of the anti-inflammatory cytokine (IL-4: 0.696 ± 0.247), when compared to NB- (IL-4: 0.390 ± 0.256; IL1-β: 10.864 ± 1.026; IL-6: 84.702 ± 38.767; IFN-γ: 9.976 ± 3.174) and SH- (IL-4: 0.606 ± 0.363; IL1-β: 4.232 ± 1.106; IL-6: 1.143 ± 1.143; IFN-γ: 2.871 ± 1.521) treated groups. (b) Representative networks of molecules present in ASC secretome, and respective receptors and signaling molecules, based on their role on inflammation and immune response (in green), and neurogenesis and axonogenesis (in blue), identified using STRINGS bioinformatics tools. in Mean ± SEM; *n* = 7 for SH and CM groups; *n* = 4 for NB group; *p < 0.05.

The pro-inflammatory IL-1β, IL-6, and IFN-γ, and the anti-inflammatory IL-4 cytokines were probed in the blood serum of SH-, NB-, and secretome-treated animals, 6 weeks post-injury (Figure 5). IL-1β was found to be decreased in the secretome-treated group in comparison to NB-group, although with no statistical significance. Moreover, IL-6 and IFN-γ were significantly decreased following secretome treatment CM-group (2.286 ± 1.460 and 1.573 ± 0.704), when compared to NB-treated group NB-group (84.702 ± 38.767 and 9.976 ± 3.174). Finally, IL-4 was increased in the secretome-treated group when compared to NB-group, but no statistical differences were found between groups. Concentrations of pro- and anti-inflammatory cytokines in the secretome-treated group were very similar to those observed in the SH-group. We further investigated the existence of possible interactions between some of the interleukins and growth factors we know being present in the secretome of ASCs [37]. We have specifically looked for interactions between IL-6, IL-1β, IFN-γ, IL-4 e IL-10, e PEDF (SERPINE1), GDN (SERPINE2), SEM7A, DCN, and β4Galt1. The analysis was performed based on their role on processes such inflammation and immune response, represented with a green tag, and neurogenesis, axonogenesis, represented with a blue tag (Figure 5).

## Discussion

In drastic contrast to humans and mammals, amphibians have a remarkable capacity to fully regenerate.^44^ Therefore, using these species as models for studying (SCI) can help to unveil the features and mechanisms by which a natural regeneration process occurs. Among these different naturally-regenerating species, *Xenopus laevis* is probably the most interesting model as it presents a stage-dependent regeneration,^45^ in which their entrance in metamorphosis initiates a progressive loss of their regenerative capacity, defining the transition from a regenerative to a non-regenerative stage. Interestingly, these animals also display a particular developmental stage in which they transiently lose this capacity, the so-called refractory period.^46,47^ The alteration of the regeneration program on *Xenopus laevis* during this period is not dependent on metamorphosis but rather on other mechanisms, such as immune^48,49^ and/or metabolic^45^ alterations, and other cellular and transcriptomic changes.^47,50–52^

In the present study, the use of ASC secretome after spinal cord complete transection in *Xenopus laevis* tadpoles favored axonal regeneration which was correlated with improved functional recovery after injury. As observed in Figure 1(c), the use of ASC secretome significantly increased levels of regenerating fibers at 2 days post-treatment (Figure 1(e)). Indeed, it supported important although not statistically significant axonal sprouting accompanied by the formation of a robust axonal bridge between the two stumps of the spinal cord at 5 days post-treatment (Figure 1(e)). According to Beck et al.,^47^ refractory period tadpoles never progressed to tissue regeneration after tail amputation.

In contrast, regenerative tadpoles showed regenerated tails, including the spinal cord, within approximately 1 week after injury. Also, complete cellular bridge formation across the lesion in regenerative tadpoles was only reported 10 days post injury by Muñoz et al. and colleagues.^53^ Others have reported similar dynamics of regeneration.^54,55^ Thus, our observations suggest that ASC secretome treatment restores the regenerative ability of the refractive tadpoles. Additionally, the recovery of the swimming ability was observed for tadpoles in this stage, after ASC secretome treatment (Figure 1 (a, b and e), respectively), in contrary to NB-treated animals, which correlated with the observed histological improvements.

Interestingly, the temporary loss of tadpoles’ regenerative ability in the refractory period has been previously attributed to a suppression of specific molecular pathways, namely BMP and Notch signaling pathways^47,56^ and a transitorily imbalanced immune response,^48^ as well as to a marked decrease of Sox2/3 positive cells at the lesion site following injury,^53,55^ all contributing differently but possibly synergistically to the decreased regenerative capabilities of these animals. Whether the ASC secretome may be acting upon these or other mechanisms was not unveiled in this study. However, we and others have extensively demonstrated that ASCs secrete factors related to neuroprotection, neurogenesis and axonogenesis.^27,29,32,34,36^ One of the first and most important hallmarks of the regenerative process of *Xenopus laevis* after injury is neurogenesis, accomplished by the recruitment of animal’s tissue resident adult stem cells to the injury site which participate in tissue regeneration and repair that follows injury.^53,55^ Enlighted by the above-demonstrated data obtained from SCI on tadpoles after treatment with ASC secretome, we believe that the secretome may have the potential to act upon the regeneration of these animals, either through a direct action upon neurogenic niches^57,58^ or by promoting the activation of specific molecular pathways or related genes.^59^

As referred above, the BMP signaling pathway has been implicated in the normal regeneration of *Xenopus laevis* after tail amputation,^46,47^ with the suppression of this pathway contributing to the loss of the regenerative ability of tadpoles in the refractory period. In the present study, we show that the treatment of these tadpoles with secretome helps the recovery of this ability, which may indicate an action of the molecules secreted by this cell population. PEDF, for example, previously identified in ASC secretome^37^ has been considered a facilitator of neurogenesis, by mediating BMP signaling pathway, specifically through the activation of Msx1-related gene has been previously implicated in the regeneration of *Xenopus laevis*.^56^ However, in this specific study, the authors claim that Msx1 activation of these animals is insufficient to promote the complete regeneration of these animals. Instead, additional BMP targets or other mechanisms may be potentiating the regeneration of these animals, indicating that there should be an interplay of different mechanisms or genetic programs.^56^ This makes sense that this same factor, PEDF, is also known to act on other signaling pathways, such as Notch,^60^ to regulate the stemness of NSCs. As the Notch signaling pathway was also found to be suppressed in the refractory period tadpoles,^47^ its activation along with the BMP signaling pathway by PEDF would largely benefit the regeneration of these animals.

The activation of BMP signaling pathway has been shown to be mediated by many other molecules, namely SMAD and STAT-3. In our previous study, these two molecules were identified as effector molecules linked to PEDF and IL-6 and Cadherin 2,^36^ two molecules also identified in ASC secretome.^37^ Specifically for STAT-3, IL-6 acts on this effector molecule by suppressing its activity, thus repressing neurogenesis.^61^ Nevertheless, IL-6 repressive role on this mechanism can be balanced by the opposite action of IL-10, which induces neurogenesis through the reactivation of both BMP and STAT3 signaling.^62^ In what regards CHD2, it is well established the role of this molecule on the neurogenesis of vertebrates,^63^ during both development^64^ and following injury,^65^ as well as on axonogenesis.^66,67^ Once again, the control over these processes requires the activation of Notch and Wnt signaling pathway by CHD2.^63,67^

Interestingly, STAT-3 has also been suggested by Foshay and Gallicano^68^ to directly regulate neurogenesis by inducing the expression of Sox2 and subsequently nestin expression of committed NPCs. The increased expression of Sox2^+^ cells mediated by ASCs has been shown by others. Oh et al. ^69^ attributed the ASC-mediated increased of Sox2 expression in NSCs through the activation of Wnt signaling pathway in a model of Alzheimer’s Disease, while Munoz et al. ^57^ demonstrated that the transplantation of BMSCs into the dentate gyrus of immunodeficient mice increased the proliferation of endogenous Sox2^+^ NSC cells, due to a local increase of VEGF, Ciliary neurotrophic factor (CNTF), NT4/5 and NGF. Therefore, there is the possibility that the secretome of ASCs might be helping tadpoles to regain their regenerative ability by recuing Sox-2 positive cells proliferation and differentiation, or activating molecular pathways elsewhere identified as crucial to give support to neurogenesis occurrence after SCI. In future, studies directed to explore the mechanisms underlying the effects of ASC secretome on the regeneration of tadpoles after SCI will be of utmost importance.

Following the positive outcomes obtained from the *Xenopus laevis* SCI model, we aimed to study whether the ASC secretome would have a therapeutic effect in an animal model with decreased levels of regeneration in the CNS, such as mice, using the same type of lesion. All the transected mice presented complete paraplegia of both hindlimbs 2 days after injury, confirming the spinal cord’s complete transection. Locomotor analysis of mice treated with ASC secretome showed a significant clear and progressive motor recovery, beginning at 2 weeks post-treatment. At this time-point, the secretome group presented the ability to perform coordinated plantar stepping. In contrast, the NB-treated group only presented slight movement of ankles (Figure 2(a)), according to the BMS score.^70^ The locomotor improvements of the secretome-treated animals were accompanied by a temporary regain of sensitivity at 2 weeks as shown by the higher magnitude of animal’s response to Von Frey filaments in the secretome-group which subsided at the sixth week. Such initial response may have risen from early regenerating sensory fibers that later mature^71^ (Figure 2(b)). Importantly, the reduction in the reaction threshold seen in the sixth week may also represent a learned behavior where filaments cease to evoke a motor response.^72^

An important factor influencing the successful regeneration of a tissue after the injury is the inflammatory response. Inflammatory cells, namely microglia and macrophages, are the significant contributors to the post-injury inflammatory response.^73^ These cells are described to rapidly respond upon injury, changing their morphology and phenotype to an activated form or classically activated microglia (M1)—a round and enlarged cell soma with retracted processes, or to an alternatively activated or pro-regenerative microglia (M2)—a ramified morphology with extended thin processes.^74,75^ The production of cytokines and growth factors by these cells provide them with either neurotoxic (M1 state), or neuroprotective (M2 state) character,^74,76–78^ although their role in the injured spinal cord is still a matter of intense debate.^79,80^ Therefore, the magnitude of an inflammatory response might be determined by the activation/deactivation state of the inflammatory cells along the spinal cord’s rostro-caudal axis after injury. In this context, our data shows a clear impact of the secretome of ASCs in the modulation of microglial cell responses following SCI. As demonstrated in (Figure 3(a)) the areas of ameboid and ramified microglia in the spinal cord are clearly shifted in the CM when compared to the NB treated group to a profile suggestive of controlled inflammatory response. It is important to note that our quantification is based on the dynamic shift of area occupancy of microglial cells with an ameboid versus a ramified morphology after SCI. In this regard the emergence of an anti-inflammatory phenotype should be interpreted by the representative rostro-caudal image and our quantification plots in (Figure 3(a) and (c)) respectively, rather than just by the close-up images, as the proliferation response of microglial reactivity is visible. Crucially, this method has been validated and employed in several publications assessing microglial reactivity and neuroinflammation after spinal cord injury.^81–84^ Together, these data suggest an attenuation of the cytotoxic impact of the inflammatory cells on the injured animals following ASC secretome treatment, possibly partially accounting for the observed improvements of locomotion and tissue regeneration, such as the increased expression of GAP-43. Importantly, it is worth mentioning that a certain degree of inflammation followed by a homeostatic resolving phase has been demonstrated to be crucial for axonal regeneration and positive functional outcomes.^85^ In similarity to what we have found here, other studies have shown that the transplantation of ASCs directly into lesion site led to increased numbers of M2 macrophages, and decreased numbers of the M1-type in a contusion model SCI.^86^ Interestingly, these changes were associated with motor function improvements, accompanied by axonal preservation, less scar tissue formation and increased myelin sparing. In this study, the beneficial effects of ASCs were attributed to increased IL-4 and IL-13 levels and reduced levels of TNF-α and IL-6, supporting an immune modulation of ASCs through paracrine actions.^86^ Other study have also shown that NGF, BDNF, or NT-3 promoted immune deactivation,^87^ and the upregulation of TGF-β, IL-4, or IL-10 downregulated microglial cytotoxicity.^88,89^ In the context of traumatic SCI, the suppression or depletion of microglia activation led to significant locomotor improvements and overall tissue integrity.^90–92^ More recently, the attenuation of inflammatory response after SCI was clearly shown by Cizkova’s study using a SCI contusion rat model. This was correlated with the modulation of pro-and anti-inflammatory molecules by the secretome of BM-ASCs, namely by decreasing IL-6 and TNF-α levels and increasing the levels of VEGF and CNTF.^93^

Along this line, the anti-inflammatory role of ASC secretome after SCI is believed to be accomplished either by the secretion of inflammation-related molecules that directly activate molecular pathways that regulate inflammatory responses that modulate microglial cell activity. Microglia and macrophage activity during inflammatory responses are highly dependent on cytokine stimuli. At the same time, M1 microglia are usually induced by pro-inflammatory cytokines such as IL-1β, IL-6, TNF- α, and IFN-γ, M2 microglia are mainly induced by anti-inflammatory cytokines such as IL-4, IL-13, and IL-10.^94,95^ Therefore, we performed a cytokine array to identify some inflammation-related cytokines in the blood serum of injured mice, namely the pro-inflammatory IL-1β, IL-6, IFN-γ and anti-inflammatory IL-4, after complete SCI transection. The levels of IL-1β, IL-6, IFN-γ were significantly decreased in the blood serum of secretome-treated animals 6-weeks post-injury, while IL-4 had a tendency to increase, compared to NB-treated animals (Figure 5). IL-1β is a subtype of IL-1 that is immediately produced by neurons and microglia in response to injury, and acts directly on the exacerbation of inflammation, together with IL-6 and TNF-α.^96,97^ Specifically, for IL-6, this cytokine expression is sharply increased in the acute stages after SCI. The suppression of its activity had regenerative effects associated with reducing glial scar formation, axonal sprouting and regeneration, and functional recovery.^98,99^

Regarding INF-γ, this cytokine is produced by astrocytes at later stages of SCI,^100^ and reported to induce the expression of other pro-inflammatory cytokines and molecules such as ROS and iNOS,^101^ therefore contributing to microglial and astrocyte activation, as well as to neuronal degeneration and neuropathic pain.^102^ On the other hand, IL-4 is a classically anti-inflammatory cytokine described to modulate microglia activity and act upon SCI-related events such as inflammation, glial scar formation, and yet provide neuroprotection.^81,103^ In fact, in the study carried out by Sato et al.,^96^ the treatment of microglia cells with IL-4 induced the expression of markers associated to M2 phenotype, suggesting an effect of this cytokine in microglia cytotoxicity regulation. Later on, microglia and macrophages polarization into a tissue repair-phenotype (M2) after intraspinal injection of IL-4 in mice following SCI contusion, supported this hypothesis.^104^ In that study, IL-4 effect led to functional recovery and decreased tissue damage after SCI. More recently, published data from our lab pointed to the same direction by showing that the systemic administration of IL-4 to rats after SCI decreased the number of inflammatory cells.^81^ The increased levels of IL-4 were related to neuronal survival, and locomotor recovery of IL-4 treated animals,^81^ going in accordance with our findings.

The presence of these and other cytokines and growth factors specifically in ASC secretome has been widely explored for their ability to regulate inflammation. The detection of TGF-β, HGF, prostaglandin E2 (PGE2) and IL-10 on the secretome of ASCs suggested that the immunosuppressive effect of these cells might be mediated by the secreted cytokines.^105^ Moreover, ASCs were found to respond to inflammatory stimuli by adjusting the secretion of hematopoietic factors such as colony-stimulating factor (G-CSF), granulocyte macrophage colony stimulating factor (GMCSF), macrophage colony-stimulating factor (M-CSF) and monocyte chemoattractant protein 1 (MCP-1), pro-inﬂammatory cytokines such as IL-6, IL-8, IL-7, IL-11, and TNF-a,^106^ and angiogenic factors such as VEGF, HGF, and IGF-1.^107^ These molecules’ secretion was associated with decreased proliferation of peripheral blood mononuclear cells,^108^ and increased monocyte migration to inflammation sites.^109,110^ The presence of such factors in ASC secretome is also indicative of this cell population’s neuroprotective character. Evidence on this were reported in a study in which ASC secretome was found to protect a PC12 cell line from excitotoxicity, through the secretion of BDNF, VEGF, and HGF.^111^ Secreted IGF-1 and BDNF were also indicated as mediators of protection and recovery in a rat model of brain ischemia.^112^

We have also recently revealed the presence of proteins involved in the regulation of inflammation, neuronal differentiation, and axonal outgrowth through a proteomic analysis on ASC secretome.^37^ In this study, Decorin (DCN) was one of the proteins expressed in the ASC secretome. This is an anti-scarring molecule that is involved in the process of fibrosis, by direct interaction with pro-inflammatory factors.^113^ For example, DCN was reported to neutralize and repress the pro-inflammatory TGF-β, thus reducing fibrotic scar.^114^ Also, DCN reduced astrogliosis and decreased scar-related elements, which further supported axonal regeneration after lesion.^115^ The proteomic analysis showed that plasma protease C1 inhibitor (C1-Inh) was another molecule identified in ASC secretome. This molecule has been described to play an important role in the suppression of inflammation in a variety of inflammatory diseases. However, the true effect in the CNS is still not, known and neither are the mechanisms upon which it acts.^116,117^ Finally, proteins like SEM7A and clusterin (CLUS) have also been identified in the ASC secretome. We have previously considered these two proteins to be supportive of DRG axonal outgrowth in vitro, while others have associated them with an immune role,^118^ which might indicate that they can be involved in different mechanisms, all ultimately contributing to tissue regeneration. The fact is that the modulation of neuroinflammation in SCI mice after ASC secretome treatment observed in this study was accompanied by axonal elongation and regeneration in the transected spinal cord, at 6 weeks post-treatment, as indicated by βIII-tubulin and GAP-43 immunostaining (Figures 3(c) and 4(e); Figures 3(c) and 4(f)).

As referred, we further looked to a possible interaction between the herein analyzed cytokines and proteins previously identified by our lab in the secretome of ASCs (Figure 5). According to the obtained network, there is a clear clustering of the investigated molecules in 2 groups, one presenting a close interaction between IL-6, IL1-β, and IFN-γ, and the other with PEDF and GDN. As expected, the interleukin-composed group was labeled to be involved in inflammation and immune response (green tag). Interestingly, IL-6 is the interactor molecule between these two groups. We have previously hypothesized whether IL-6 would also have a role in axonal outgrowth. Considering the direct interaction between IL-6 and PEDF, a factor closely related to axonogenesis and neurogenesis,^26,30^ it is possible that IL-6 might be having some axonogenesis-related role through this way. Additionally, PEDF presents an inflammation and immune-related tag (green), suggesting a role of PEDF on these processes as well, and was shown to interact with GDN through the ECM glycoprotein vitronectin (VTN), PLG (uPA, urokinase-type plasminogen activator) and PLAU (plasminogen) molecules, which are components of the fibrinolytic system.^119^ These molecules have long been suggested to be involved in axonal growth and tissue remodeling in several CNS diseases,^120–122^ possibly explaining its interaction with GDN. PLG and PLAU have also been described to contribute to the inflammatory response in several CNS inflammation and demyelination contexts, usually closely related to activation of matrix metalloproteinases (MMPs).^123^ For example, in multiple sclerosis, PLG and its receptor were highly concentrated on inflammatory cells in acute lesions, facilitating its infiltration into the CNS through the action of specific MMPs, namely MMP9 and MMP1.^123^

No interactions were found between SEM7A and the other molecules in the molecular network. However, as suggested in our previous study, this semaphorin predominates neurogenesis and axonogenesis.^36^ Interestingly, SEM7A was also identified as an effector molecule for inflammatory processes (yellow tag). This molecule has been previously identified as an effector molecule in T-cell mediated inflammation, mostly through the regulation of integrins,^124^ and has been linked to nerve regeneration,^125^ supporting the indication obtained from the molecular network. Here, the plexin-C1 (PLXNC1) has been the effector molecule linked to SEM7A, which goes following studies showing an important role of PLXNC1 during acute inflammatory response.^126^ Finally, no interactions were found with DCN and β4Galt1 with the other investigated molecules in the performed analysis.

Considering all the previously discussed data, it is more likely that axonal outgrowth and regeneration result from the observed modulation of the inflammatory response following injury. Indeed, a synergistic effect of several proteins and growth factors might be protecting the spinal cord tissue from further damage at early stages post-injury (neuroprotection). Moreover, it is worth to note that the regeneration of an adult tissue is normally due in shortly after the beginning of the regenerative process.^127,128^ Therefore, the presence of GAP-43^+^ axons at the end of this experiment would not be expected. However, our data seems to suggest that the secretome of ASCs extended the regenerative process up to 6 weeks post-injury, which goes in accordance with previous indications that the exogenous supply of trophic support can stimulate GAP-43^+^ axons over longer periods.^128,129^ Along wi3th axonal sprouting and regeneration, decreased lesion cavities were also observed for the secretome-treated animals, in comparison to the NB-treated animals (Figure 4 (e) and (f)) and (Figure 3(d)). Also, it is important to highlight that further studies using intermediate time-points after SCI to investigate the relationship of ASC secretome treatment and the response of pathophysiological and neuro-restorative hallmarks are warranted. Considering this, the anti-inflammatory cytokines and regenerating growth factors provided by the continuous administration of secretome of ASCs to the SCI mice may have contributed to the overall tissue repair, adding to the observed improvements in locomotor function of these animals, as well as to the recovery of their hindlimb sensitivity. Finally, future mechanistic explorations to improve the neuroregulatory profile of ASCs secretome for SCI applications such as the manipulation of culture conditions, use of computer-controlled bioreactors or the employment of different molecular priming strategies will be instrumental in the pursuit of clinical translation.

## Conclusions

Overall, this work’s findings indicate the positive effects exerted by the secretome of ASCs on promoting functional recovery in two models of SCI. This recovery was associated with robust presence of regenerating neuronal fibers at injury site in both models. Additionally, the mouse study revealed important secretome effects in the induction of axonal outgrowth and modulation of the inflammatory response both at the site of injury and at a systemic level. It should be emphasized that the impact of ASC secretome in neuroinflammation is of extreme importance, as the inflammatory response after injury is one of the main processes that contributes to the exacerbation of the lesion. Likewise, axonal outgrowth/regeneration is also essential to restore the normal SC function.^11^

## Materials and methods

### Adipose tissue-derived mesenchymal stem cells (ASCs)

#### ASC culture

ASCs were kindly provided by professor Gimble (Lacell, USA). After thawing, cells were cultured in alpha-minimum essential medium (α-mem, Invitrogen, USA) supplemented with sodium bicarbonate (NaHCO_3_; Merck, USA), 10% (v/v) of fetal bovine serum (FBS; Biochrom, Germany) and 1% (v/v) penicillin-streptomycin antibiotic (p/s; Invitrogen, USA). When confluent, cells were enzymatically dissociated with 0.05% (v/v) trypsin/EDTA (Invitrogen, USA), re-plated at a density of 4000 cells/cm^2^ and maintained at 37°C, 5% humidified CO_2_, 95% air and 90% relative humidity.

### Conditioning and secretome collection

The secretome, denoted as conditioned media (CM), was collected from cells in passage 5, as previously described.^36^ Cells were plated at a density of 4000 cells/cm^2^ and maintained in culture for 72 h. Cells were then washed 5 times with phosphate buffered saline (PBS) without Ca^2+^ and Mg^2+^ (Invitrogen, USA), and 1 time with the conditioning medium—Neurobasal A Medium supplemented with 1% (v/v) Kanamycin (Invitrogen, USA). After 24 h of conditioning period in supplemented Neurobasal-A Medium, the secretome was collected and centrifuged to remove cell debris. The collected secretome was concentrated 100x using a Vivaspin 20 centrifugal concentrators (MWCO 5 kDa, Sartorius™ Vivaspin™ 20, Germany) at 3000*g*, and frozen at −80°C until further required.

### *Xenopus laevis* in vitro fertilization, eggs maintenance, and follow-up

#### In vitro fertilization

Female frogs were primed with human chorionic gonadotropin (HCG) 5–14 days before the experiments. 500–800 units of HCG was injected in the female frog’s lymph sac approximately 12 h before the eggs were needed. In the fertilization day, female frogs were placed for egg laying into 1x Modified Barth Buffer solution (MBS, 10x MBS salts [51.3 g NaCl, 0.75 g KCl, 2.0 g MgSO_4_ × 7H_2_0, 23.8 g HEPES, 2.0 g NaHCO_3_], 0.1 M CaCl_2_ and 5 M NaCl in 1L distilled H_2_O). Eggs were carefully collected regularly into 1x MBS into 100 mm petri-dishes. Meanwhile, male frogs were anesthetized in 0.1% (w/v) tricaine methanesulfonate (MS222) for 15–30 min until slow or no heartbeat was found. Male’s testes were excised and kept on ice. Small pieces of testis were macerated and mixed with the collected eggs into 0.1x MBS for 20 min at room temperature (RT). A cysteine treatment was then performed to remove egg’s jelly coat by incubating them with 2% (v/v) Cysteine solution in 0.1x MBS for 5–7 min at RT with gentle rocking. Eggs were washed for 5–10 min with 0.1x MBS and transferred to a cold plate at 14°C, or at RT for approximately 1 h until the first division occurs and then transferred to 14°C.

#### Eggs maintenance and follow-up

Dead eggs were removed from the petri dish twice a day using a pipette, and 0.1x MBS was replaced. The developmental stages of the animals were followed every day, using the developmental data from Nieuwkoop and Faber.^130^

### Spinal cord injury infliction and post-operative care

#### Xenopus laevis model

**Figure 6.**
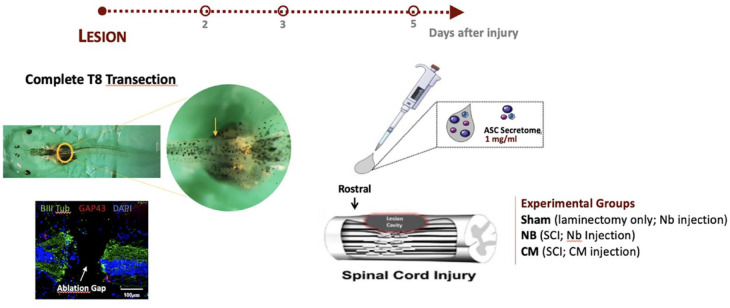
Schematics of the experimental paradigm employed for the *Xenopus laevis* model of transection SCI.

Tadpoles in the stages 45–47 (refractory period) were used in this work. For that, animals were closely checked daily for their developmental stages. When the desired stage was reached,^131^ animals were carefully collected and used for the experiments. A complete transection of the spinal cord of *Xenopus laevis* was the injury model used in this work as shown in (Figure 6). To inflict the transection into the animal’s spinal cord, the protocol described by Edwards-Faret et al.^132^ was used as reference. Briefly, tadpoles were anesthetized with 2% (w/v) of freshly prepared MS222 by immersing the animals in the solution for 1–2 min. Animals were then carefully immobilized on their abdominal area using forceps under a dissecting microscope. A small incision on the skin and dorsal muscles perpendicular to the body’s axis was made at the mid-thoracic level of the animals at the gut’s central level. The meningeal layer was then removed using forceps to completely expose the spinal cord.^132^ At this stage, animals were grouped according to the procedure/treatment to receive: (1) non-injured tadpoles, injected with Neurobasal-A (SH group; *n* = 12); (2) tadpoles subjected to SCI, injected with Neurobasal-A medium (NB group, *n* = 12); and (3) tadpoles subjected to SCI, injected with ASC secretome (CM group, *n* = 12). To fully transect the spinal cord of the tadpoles, a tip of a 30-gauge needle was used to make a clean cut at the thoracic level, perpendicular to the spinal cord. A successful spinal cord transection was confirmed by checking the presence of a dark line between rostral and caudal stumps of the spinal cord.^132^ After surgery, all animals were transferred to small dishes containing 1x MBS and antibiotics [penicillin (5000 U/ml)-streptomycin (5 mg/ml) solution (Sigma-Aldrich, Germany) and gentamycin (1.25 mg/ml, Fisher Scientific, UK)], defined as 0.1x MBS + 3A, and kept at 20°C–21°C until they recovered from anesthesia. The post-operative care of the animals with the antibiotics was maintained for 3 days, with the 0.1x MBS + 3A solution being replaced twice a day.

#### Mice model

**Figure 7.**
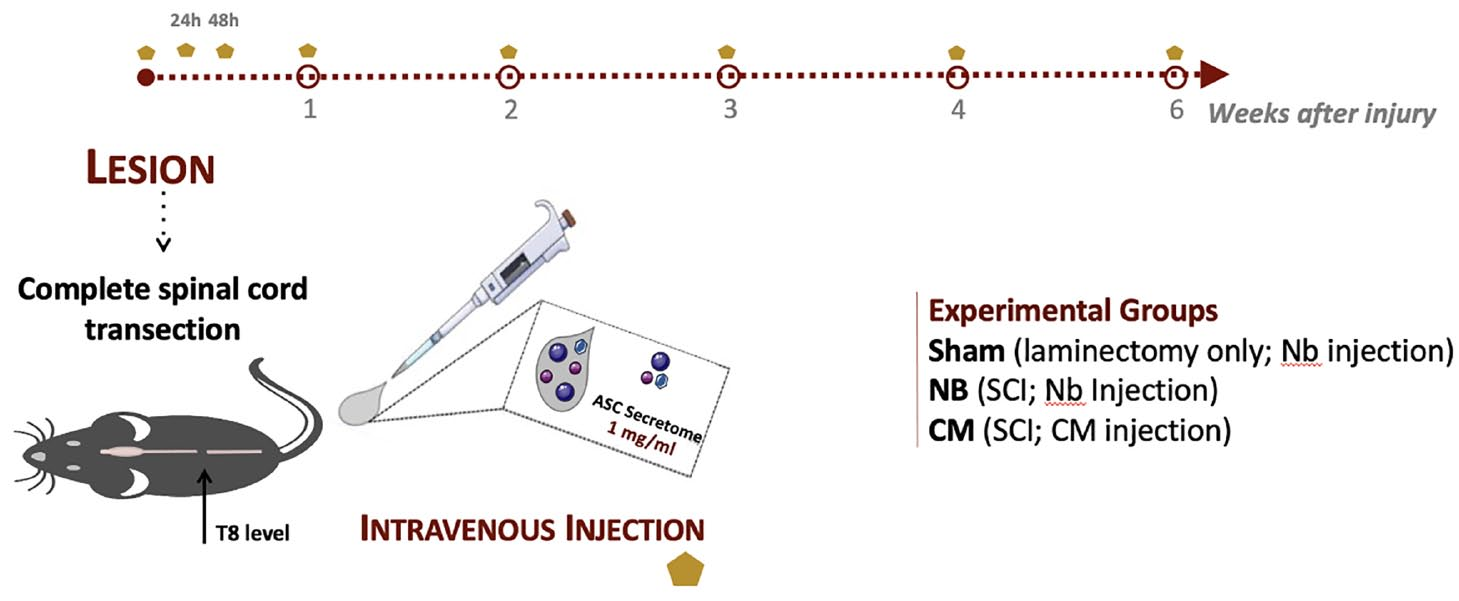
Schematics of the experimental paradigm employed for the mouse model of transection SCI.5.5. Secretome injection in the Xenopus tadpole’s spinal cord after injury.

Eight-week-old female C5Bl/6 mice (Charles River, France), were used in this in vivo study. Animals were group housed—5 per cage, on corncob bedding with access to food and water ad libitum, and holding rooms were maintained on a 12-h light/dark cycle. A complete transection of the spinal cord was the injury model herein used as outlined in (Figure 7). Briefly, animals were anesthetized with a mixture of 1.5x Imalgene and 1x Dorbene. When no reaction to pinch was observed, animals were considered ready for surgery. First, animals were placed under a dissecting microscope. An incision on the skin and dorsal muscles was performed from T2 to T10 and the muscles retracted. A laminectomy was performed at the T8 level, and the spinal cord exposed. At this stage, animals were grouped according to the procedure and/or treatment to receive: (1) mice subjected to sham operations—laminectomy but no SCI, injected with Neurobasal-A medium (SH group, *n* = 8); (2) mice subjected to SCI, injected with Neurobasal-A medium (NB group, *n* = 7); and (3) mice subjected to SCI, injected with ASC secretome (CM group, *n* = 9). The spinal cord of NB and secretome group animals was totally cut using a microdissection scissor. The complete separation of both ends of the spinal cord was confirmed under the microscope using forceps. Animals were finally closed with Vicryl sutures (Johnson and Johnson, USA). After the surgical procedure, anesthesia effect was reverted by a single subcutaneous administration of atipamezole (5 mg/ml, Antisedan/Pfizer, USA). Post-operative care consisting in subcutaneous administration of the analgesic butorphanol (10 mg/ml, Butamidor, Richter Pharma AG, Austria), the antibiotic enrofloxacin (5 mg/ml, Baytril/Bayer, Germany), 0.9% (v/v) NaCl and vitamins (Dulphalyte, Pfizer) was then given to every animal. Animals were then kept under heat lamps until recover from anesthesia. Post-operative care was maintained twice a day for 1 week post-injury. Manual bladder voiding was performed twice a day until animals recovered their bladder control completely. The general health of the animals was carefully checked every day for signs of illness and weight loss, during the time of post-surgery recovery and treatment.

#### Local injection of ASC CM

The secretome of ASCs was injected locally in the rostral stump of the spinal cord, immediately after injury. For that, a Pneumatic Pico Pump System (Narishige Group, Tokyo, Japan) was used. First, a pulled-glass capillary needle was filled with 2 μl of the labeled secretome. The needle was then set in the needle holder, and the tip of the needle was carefully placed on the ependymal canal, rostral to the spinal cord, with the help of the micromanipulator under the dissection microscope. 2 μl of secretome was administered at the tadpole’s ependymal canal of the spinal cord at a rate of 0.05 μl/s. NB group animals were injected with Neurobasal-A medium.

### Secretome administration to mice after SCI

After injury, animals from the CM group were intravenously administrated with ASC secretome. The treatment was given systemically to the animals in the three 24 h post-injury, the first immediately after SCI, and then weekly until 6 weeks post-injury (100*µ* l per injection). SH and NB group animals were administered with 100l of Neurobasal-A medium.

### Swimming behavior of Xenopus laevis tadpoles

To evaluate the effect of ASC secretome on the functional recovery of the animals after injury, their free-swimming behavior was analyzed at 2, 3, and 5 days post-injury. Animal’s swimming trajectory was evaluated using a custom-made optimized vibrating six-well plate along with a video-tracking system (DanioVision, Noldus, Netherlands). For that, animals were individually placed in wells of a six-well plate containing vibrating motors attached to their walls (Pico Vibe™ 10 mm Vibration Motors (Precision Microdrives, UK)), and their movement was tracked and recorded by a camera inside the DanioVision chamber. The parameters of the test were set using the Ethovision software (Noldus, Netherlands). The animals were left to acclimatize without disturbance for 10 min before testing. Once the vibration mode was set to ON, animals were subjected to cycles of 4 s of vibration, followed by 12 s of resting period (no vibration), to a total of 80 s of test. The recording data of the animals was acquired by the EthoVision software.

### Motor and sensorial behavior of mice after SCI

#### BMS score

Basso Mouse Scale (BMS) scoring was used to assess the locomotor recovery of secretome-treated mice after SCI.^70^ The first BMS evaluation was performed 2 days after injury to confirm hindlimbs paraplegia in all animals. Scores of 0 were selected for the experiment. BMS scoring for locomotion evaluation was then performed weekly until the end of the experiment (6 weeks post-injury). Mice were allowed to explore an open field arena for 5 min, while their locomotion was being recorded by video-camera. Two blinded researchers evaluated mice locomotion during the entire duration of the trial.

#### Von-Frey test

The ability of secretome-treated SCI mice to respond to a mechanical stimulus at the hindlimb paws was assessed by the Von Frey test.^43^ For that, mice were individually placed in a clear glass in an elevated grid and the plantar surface of the hind paws was poked with Von Frey filaments of varying forces—2*g*, 1.4*g*, 1*g*, 0.6*g*, 0.4*g*, 0.16*g*, 0.07*g*, 0.04*g*, 0.02*g*, and 0.008*g*. The trial started using the middle force filament (0.16*g*) and went further up the higher force filaments in case of no reaction (=0), or down to the lower ones in case a reaction occurred (=X), in a total of 6 measurements.^133^ If no response was obtained up to the maximal filament (2*g*), or if a positive response occurs down to the minimal (0.008*g*), the 1.4 and 0.008 values were assumed to measure that animal, respectively. Positive reactions considered included paw withdrawal, licking, shaking or extension of the paw, either during the stimuli or immediately after. The presence of nociception or hypersensitivity on the hindpaws was indicated by an exaggerated reaction to the lower diameter filaments. The response to Von Frey filaments was deduced as the 50% response threshold, calculated using the formula $50\%g_{threshold}=\frac{10^{Xf+K.\delta }}{10000}$ , where X is the value corresponding to the final Von Frey filament tested (in log units); k is the tabular value concerning the pattern of positive (=X) and/or negative (=0) responses, and δ is the mean difference between stimuli (in log units).^134^ Low threshold indicates hypersensitivity, while a high threshold indicates normal sensitivity to the mechanical stimuli, usually found among healthy individuals.^43^

### Histological preparation of the animals

#### Xenopus laevis model

Tadpoles were sacrificed by terminal anesthesia by immersion into 2% (w/v) MS222 for 15 min, and placed in 4% (w/v) of paraformaldehyde (PFA) solution for 1 h at RT. Animals were washed 3 times with 1x PBS, and placed on a solution of sucrose at 30% (w/v). After 24–48 h, animals were carefully immersed in section medium (Neg-50, Thermo Scientific, USA), frozen in liquid nitrogen, and stored at −20°C. Later on, longitudinal cross sections of 20*µ*m thickness were taken using a Leica CM1900 cryostat and kept at −20°C until required for immunohistochemistry.

#### Mice model

Six weeks after SCI, mice were deeply anesthetized by intraperitoneal injection of sodium pentobarbital (200 mg/ml, Eutasil, Ceva Saúde Animal, Portugal), and transcardially perfused with 0.9% NaCl followed by cold 4% (w/v) PFA. The spine and the spinal cord were dissected and incubated with PFA for 24 h, at 4°C. The spinal cord was then carefully dissected and placed on a solution of 30% (w/v) sucrose for 24 h at 4°C. After that, 3 cm length of spinal cord tissues were cut having the lesion site at the middle point, carefully immersed in section medium (Neg-50, Thermo Scientific, USA), frozen in liquid nitrogen, and stored at −20°C. Longitudinal cross sections of 20 *µ*m thickness were then taken using Leica CM1900 cryostat and kept at −20°C until required for immunohistochemistry.

### Immunohistochemistry (IHC)

#### Xenopus laevis model

Tadpole’s spinal cord sections were immunostained for axonal growth and regeneration [βIII-Tubulin and growth associated protein (GAP)-43, respectively]. For that, sections were first washed with 0.1% (v/v) of Triton-X 100 (Sigma) in 1x PBS (PBS-T), 3 times, for 5 min to remove the excess of frozen section medium. Sections were then incubated with 3% (v/v) PBS-T for 10 min for permeabilization of the tissue. After that, tissue sections were blocked with a solution of 3% (w/v) bovine serum albumin in PBS-T for 1 h at RT to avoid unspecific binding of the antibodies. Next, sections were incubated for 1 h with the following antibodies: mouse monoclonal Acetylated Anti-Tubulin (1:500, Sigma, Cat. # T6793) and rabbit anti-GAP-43 (1:500, Abcam, Cat. # ab12274). Sections were then exposed for 1 h at RT to the respective secondary antibodies: Alexa Fluor 488 rabbit anti-mouse and Alexa Fluor 594 goat anti-rabbit (1:500; Invitrogen). Finally, all sections were counterstained with DAPI (4′,6′-diamino-2-fenil-indol; 1 mg/ml, Invitrogen) for 5 min at RT. 3 washes with 1x PBS were performed between steps. The sections were mounted in Immu-Mount (Thermo Scientific, USA) and imaging of the tissue was performed using a confocal point-scanning microscope (Olympus FV1000) at 20x magnification.

#### Mice model

Mouse spinal cord sections were immunostained for axonal growth and regeneration [βIII-Tubulin and growth associated protein (GAP)-43], de/re-myelinization (Fluoromyelin) and neuroinflammation [Ionized calcium binding adaptor molecule 1 (Iba-1)]. For that, sections were permeabilized with 0.2% (v/v) PBS-T for 10 min, and washed 3 times with PBS 1x. All sections were incubated with a blocking solution of 5% (v/v) fetal calf serum in 0.2% (v/v) PBS-T for 30 min at RT, and incubated overnight at RT with the following antibodies: rabbit anti-beta III tubulin (1:1000, Abcam), mouse anti-GAP-43 (1:1000, Abcam), rabbit anti-Iba1 (1:750, Wako). Sections were then incubated for 1 h at RT with the following respective secondary antibodies: Alexa Fluor 488 rabbit anti-mouse, and Alexa Fluor 594 goat anti-rabbit and rabbit anti-mouse (1:1000; Invitrogen). Cell nuclei was counterstained with DAPI (4′,6′-diamino-2-fenil-indol) for 10 min. For Fluoromyelin staining, sections were incubated with the FluoroMyelin™ Green Fluorescent Myelin Stain (1:300, ThermoFisher), along with DAPI, for 10 min at RT. 3 washes were performed between steps. The sections were mounted in Immu-Mount (ThermoFisher Scientific, USA) and kept at 4°C until imaged. βIII-Tubulin, GAP-43 and Fluoromyelin were imaged by fluorescence microscope (Olympus BX61) at 10X magnification, and Iba-1 immunostained sections by confocal point-scanning microscope (Olympus FV1000) at 20X and 40X magnification. All images from both models were processed and analyzed using Image J software and are displayed according to the rostro caudal orientation legends provided throughout the panels.

### Tissue histological analysis

#### Xenopus laevis model

Quantification of βIII-Tubulin and GAP-43 immunoreactivity was performed on epicenter segment of the spinal cord, in both injured secretome-treated (CM group) and non-treated (NB group) animals. Using the Image J software, all acquired images were converted into monochrome 8-bit images. Fluorescent particles appear as black pixels, and background as white pixels. The region of interest (ROI) to analyze was determined considering the total area occupied by the lesion core, defined using the free-hand drawing tool, and 500 µm rostrally and caudally from the limits of the lesion core. The expression of immunofluorescence within the ROI was evaluated automatically by the software. Mean values within each group were calculated as the percentage of βIII-tubulin- or GAP-43-expressing axons, normalized to the total segment of the spinal cord analyzed.

#### Mice model

βIII-tubulin, GAP-43, Fluoromyelin and Iba-1 reactivity was quantified on rostral, epicenter and caudal segments of the spinal cord, for both secretome and NB groups. The process of image analysis was performed using Image J software. Thus, mean values of immunofluorescence within the region of interest (ROI) defined were calculated as the percentage of βIII-tubulin^+^ or GAP-43^+^ axons, per group. Fluoromyelin staining was used to quantify the demyelinated lesion areas of the spinal cord, for both secretome and NB groups. For that, the ROI to be analyzed was determined considering the total area occupied by the lesion epicenter, defined using the free-hand drawing tool, and 500 µm rostrally and caudally from the limits of the epicenter. The corresponding area was automatically calculated by the software.

Finally, Iba-1 immunoreactivity was used to compare the area occupied by activated and deactivated inflammatory cells throughout the spinal cord tissue of the secretome and NB groups. Activated and deactivated microglia were identified within the spinal cord tissue through analysis of cell’s morphology, considering that activated microglia presents a round and enlarged cell soma with retracted processes, and resting microglia shows a ramified morphology with extended thin processes. Within the graphs, activated and deactivated microglia were considered as activated and resting microglia.

Following these considerations, the ROI and the mean values of immunofluorescence within the ROI for both activated and resting microglia were calculated as the percentage of Iba-1 reactivity.

βIII-tubulin, GAP-43 and Iba-1 measures were normalized to the total segment of the spinal cord analyzed.

### Cytokine Array

The presence of pro- and anti-inflammatory cytokines in the blood serum of mice after injury and treatment with ASC secretome was assessed using a multiplex-based ELISA. For that, blood samples were collected from the tail of the animals, after 4 days of the last CM injection, and allowed to clot for 30 min and centrifuged at 10,000*g* for 10 min. Serum was then collected and frozen at −20°C until further used. An enzyme-linked immunoabsorbent assay (Millipore, USA) for IL1-β, IL-6, IFN-γ, and IL-4 was performed following supplier’s instructions. Samples were read at Bio-Plex MAGPIX Multiplex Reader and the data analyzed using the Bio-Plex Manager™ MP Software (Bio-Rad Laboratories, Lda, USA).

### Statistical analysis

The data obtained from behavioral analysis and tissue fluorescence quantification analysis were reported as Mean ± SEM throughout the text with further information in each figure legend. The assumption of normality was tested for all continuous variables through evaluation of the frequency distribution histogram, the values of skewness and kurtosis, and through the Shapiro–Wilk test. Mean comparisons between two groups were tested with unpaired *t*-test. Statistical differences among three groups were assessed by One- or two-way ANOVA and Tukey’s post hoc comparisons tests, using GraphPad PRISM software (version 5.00). A *p*-value of ⩽0.05 (95% confidence level) was set as statistical significance criteria. Significant values were denoted with * for *p* < 0.05, ** for *p* < 0.01, *** for *p* < 0.001, and **** for *p* < 0.0001.
